# Oral vs. pharyngeal dysphagia: surface electromyography randomized study

**DOI:** 10.1186/1472-6815-9-3

**Published:** 2009-05-21

**Authors:** Michael Vaiman, Oded Nahlieli

**Affiliations:** 1 Department of Otolaryngology, Assaf Harofeh Medical Center, affiliated to the Sackler Faculty of Medicine, Tel Aviv University, Tel Aviv, Israel; 2 Department of Oral and Maxillofacial Surgery, Barzilai Medical Center, Ashkelon, Israel, and Hebrew University-Hadassah School of Dental Medicine, Jerusalem, Israel

## Abstract

**Background:**

A clear differential diagnosis between oral and pharyngeal dysphagia remains an unsolved problem. Disorders of the oral cavity are frequently overlooked when dysphagia/odybophagia complaints are assessed. Surface electromyographic (sEMG) studies were performed on randomly assigned patients with oral and pharyngeal pathology to evaluate their dysphagia complaints for the sake of differential diagnosis.

**Methods:**

Parameters evaluated during swallowing for patients after dental surgery (1: n = 62), oral infections (2: n = 49), acute tonsillitis (3: n = 66) and healthy controls (4: n = 50) included timing and amplitude of sEMG activity of masseter, infrahyoid and submental muscles.

**Results:**

The duration of swallows and drinking periods was significantly increased in dental patients and was normal in patients with tonsillitis. The electric activity of masseter was significantly lower in Groups 1 and 2 in comparison with the patients with tonsillitis and controls. The submental and infrahyoid activity was normal in dental patients but infrahyoid activity in patients with tonsillitis was high.

**Conclusion:**

Dysphagia following dental surgery or oral infections does not affect pharynx and submental muscles and has clear sEMG signs: increased duration of a single swallow, longer drinking time, low activity of the masseter, and normal range of submental activity. Patients with tonsillitis present hyperactivity of infrahyoid muscles. These data could be used for evaluation of symptoms when differential dental/ENT diagnosis is needed.

## Background

For decades the investigation of dysphagia has been concentrated on evaluation of single and separate swallows of normal subjects and neurological or ENT patients [1-5]. The same tendency is traced in research activities with EMG evaluation of deglutition [6-9]. Dysphagia, or difficulty with swallowing, is defined as any defect in the intake or transport of endogenous secretions and nutriments necessary for the maintenance of life [2,3]. Odynophagia is a painful swallowing and can occur even when "the intake or transport of endogenous secretions" is not affected [2,3]. While dysphagia can be with or without pain, odynophagia it its turn can produce dysphagia secondary to odynophagia as patients trying to reduce pain change their normal swallowing patterns. When a general practitioner or a family doctor consults a patient who presented complaints on difficult swallowing or painful swallowing, they usually refers him/her further to a neurologist and/or otolaryngologist. Dysphagia and odynophagia, however, are common symptoms in oral medicine as well. They can appear following dental extraction [10], bimaxillary osteotomy [11], odontogenic infection [12], and other dental problems including oral cavity oncology [13]. The connection between dentistry and dysphagia becomes more prominent in the elderly [14]. In addition to that, salivary gland diseases like benign salivary gland tumours, sialolithiasis, sialadenitis, strictures and kinks, ductal polyps, etc. can start with pain or difficulties in mastication and swallowing [15,16].

In cases of dental surgery or oral infections, odynophagia and dysphagia are usually related [17,18]. I.e. while in cases of dysphagia of neurological origin patients do not feel pain, in cases when oral cavity is involved dysphagia frequently appears as a result of odynophagia. Thus, in fact, three conditions can be observed in patients: 1) "pure painless dysphagia" of anatomic, obstructive or neuromuscular etiology; 2) "pure odynophagia", when a patient experience pain during deglutition but the quality of swallowing itself is not affected and there are no secondary symptoms like weight loss, wheezing or pulmonary infections; 3) combined dysphagia/odynophagia when a patient feels pain during swallowing and swallowing itself is impaired.

Instrumental investigations like barium esophagram, manometry, manofluorography, bolus scintigraphy, videofluoroscopic swallowing study (VFSS) and videoendoscopic swallowing study (VESS) are essential for confirmation of dysphagia [19,20]. These options are not specific for muscle testing and have nothing to do with cases of dysphagia of oral origin with an exception of VFSS that allows clear imaging of tongue movement. Moreover, they hardly can be called as simple noninvasive and inexpensive tests. The single swallow and continuous drinking tests usual for routine surface electromyography (sEMG) investigation of deglutition might be important not only in evaluation of dysphagia, but also in evaluation of odynophagia (for example, water drinking test after tonsillectomy) and in differential diagnosis in cases of dysphagia of unknown origin.

While it is well known and generally accepted that reduced buccal tension, poor dentition, disorders involved in muscles of mastication, reduced labial closure, reduced oral sensitivity, altered tongue contour and other similar oral/dental problems can cause dysphagia [10-13,15], this knowledge is infrequently used for differential diagnosis when dysphagia is suspected. In addition to that, a referred pain is to be taken into account, so that pain originating in one area would be interpreted as coming from the other and produce secondary dysphagia. Thus, the need for a clear differential diagnosis between oral and pharyngeal dysphagia remains an unsolved problem. The current article focuses on sEMG description of swallowing in patients with predetermined oral and pharyngeal pathologies aimed to provide data for diagnosis and monitoring of dysphagia/odynophagia and to differentiate oral cases of dysphagia from pharyngeal cases.

## Methods

### Subjects

The patients were recruited and studied across a 14-month period (Table 1). The study was based on Ethical Principles for medical research involving Human Subjects (WMA Declaration of Helsinki) and was approved by the Medical Center Ethics Committee. Informed consent was obtained from all the patients involved. Randomization was performed separately for each Group. The group of dental surgical patients (Group 1) who underwent unilateral lower second or third-molar surgery (extractions) included 62 adults, 34 women and 28 men, ranging in age from 18 to 73 years (mean = 30.1 years). This group was randomly chosen by a sealed envelope method from 217 dental patients with similar diagnosis. The group of patients with oral infections (Group 2) included 49 adults, 27 women and 22 men, ranging in age from 18 to 70 years (mean = 31.5 years). The patients in this group suffered from candidiasis (symptomatic) (n = 33) and gingivostomatitis (n = 16). This group was randomly chosen by a sealed envelope method from 197 patients with similar diagnosis. Asymptomatic candidiasis was an exclusion criterion for this group. The group of patients with acute tonsillitis (Group 3) included 66 adults, 33 women and 33 men, ranging in age from 18 to 52 years (mean = 25 years). This group was randomly chosen by a sealed envelope method from 213 outpatients with similar diagnosis. Altogether 239 patients were randomly chosen from a cohort of 627 patients. The control group (Group 4) included 50 healthy adults (age mean = 27.3 years). Enrollment procedure and participant flow is given below. Before the study all subjects completed a questionnaire regarding their general health and their medical history. The subjects had no history of dysphagia or odynophagia prior dental surgery or oral or pharyngeal infection (exclusion criterion for Groups 1, 2, 3), and no history of medical problems or medications that might affect swallowing and drinking (exclusion criterion for the controls). All subjects in Groups 1, 2 and 3 complained on difficult swallowing (inclusion criterion: food/liquid dysphagia). All subjects had normal oral anatomical structures and complete dentition with an exception of operated site (dental patients). None of patients and healthy volunteers had a history or symptoms of abnormality or disease of temporomandibular joint or any respiratory diseases, which might affect breathing (exclusion criterion). All subjects were assessed by the dental surgeons and ENT physicians prior to their participation to the study. All the males were well shaven. At the time of the test the subjects reported they were not thirsty.

**Table 1:** Enrollment procedure and participant flow (Groups 1–3).

Randomly assessed for eligibility (n = 239)			
Excluded (n = 32)			
a. Not meeting inclusion criteria (had dysphagia and/or odynophagia due to reasons other than tonsillitis or dental problems; had chronic pain type syndromes requiring pain medication) (n = 19)			
b. Refused to participate (n = 13)			
**Total allocated (n = 207)**			
Allocated to Group 1	(n = 71)	Group 2 (n = 60)	Group 3 (n = 76)
Lost to follow-up	(n = 9)	(n = 11)	(n = 10)
Analyzed	(n = 62)	(n = 49)	(n = 66)

### Electromyographic techniques

Three muscle locations were examined in the study: (1) m. masseter, (2) the submental-submandibular muscle group that includes anterior belly of digastrics, mylohyoid, and geniohyoid, and (3) infrahyoid muscle group, all covered by platisma. These muscles were selected because they are superficial and they are thought to be involved in the oral and pharyngeal phases of the swallow.

The equipment used for the EMG recordings was a NeuroDyne Neuromuscular Sys/3 four channel computer based EMG unit with NeuroDyne Medical software, and AE-204 Active sensors attached to AE-131 electrodes. (NeuroDyne, Cambridge, MA, USA). Its specifications and sEMG techniques were described in detail in our previous articles [21-23]. The computer program indicates mean, standard deviation, minimum, maximum, range of muscle activity during each trial, and its duration. Muscle activity (EMG) is quantified in microvolt RMS.

**Figure 1:**
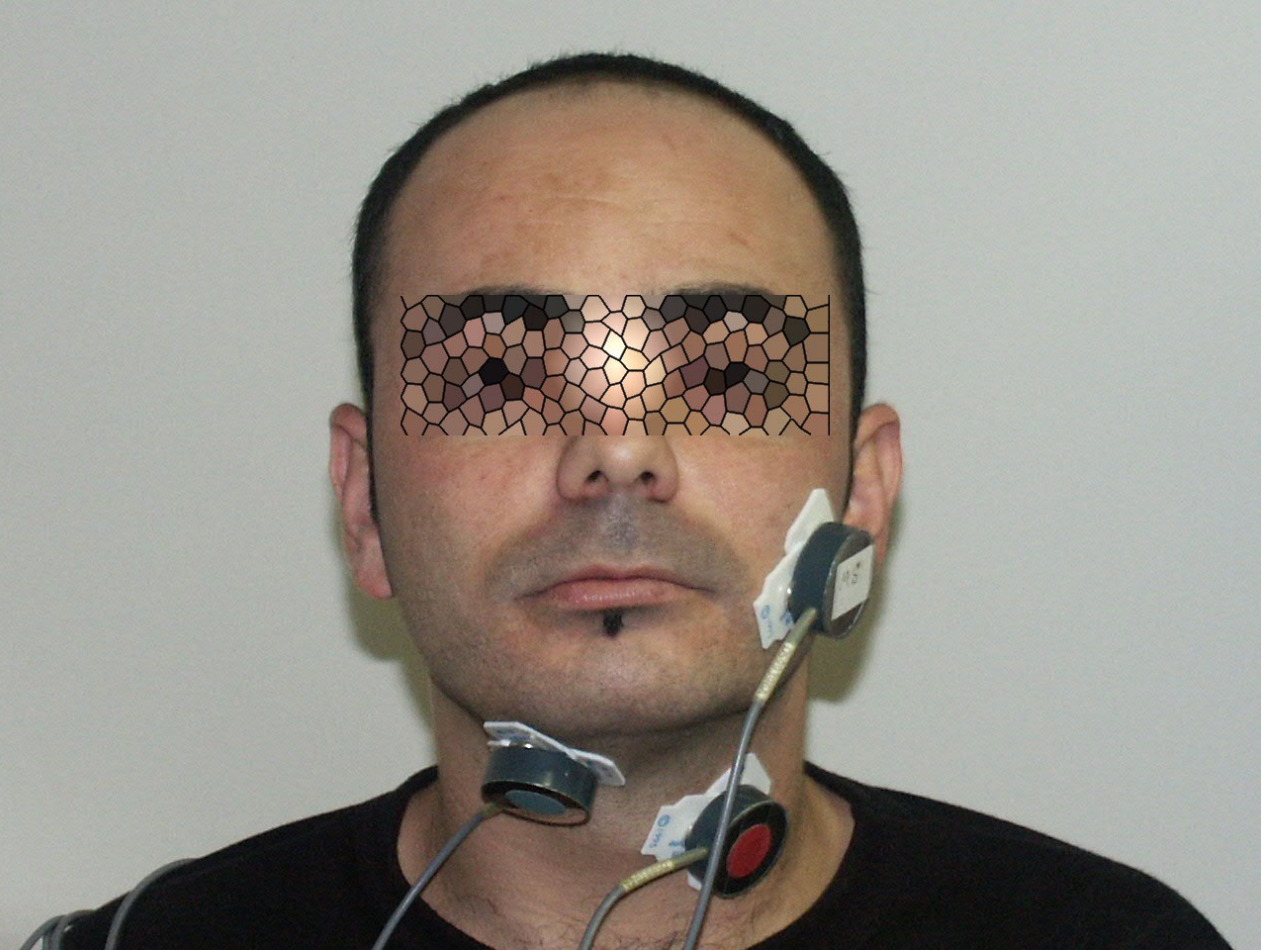
**A subject with masseter, submental-submandibular and infrahyoid muscles locations of EMG electrodes**.

The interelectrode distance was 10 mm (three electrodes – two bipolar and ground – are fixed in one stick-on patch). Specific electrode positions were as follows (Fig. 1): (1) Two bipolar stick-on surface electrodes were placed parallel to the masseter muscle fibers (MS-location). (2) Two surface electrodes were attached to the skin beneath the chin aside of midline to record submental myoelectrical activity over the platysma (SUB-location). (3) Two electrodes were placed aside of the thyroid cartilage to record from the infrahyoid and laryngeal strap muscles (INF-location).

### Procedures

The pain in odynophagia cases was assessed by visual analogue scale (VAS) pain score (0–10). Two tests were performed: voluntary single water swallows as normal from an open cup and continuous drinking of 100 cc of tap water from an open cup. In the Group 1 the tests were examined 24 hours after surgery following routine follow-up. The tests were described in detail in our previous articles [21,23]. The single swallow test was performed after a mean volume of the normal swallow bolus was calculated for group of healthy volunteers (control group).

After electrode placement, each participant performed two tasks:

1. Three trials of swallowing mean volume of tap water from an open cup. The volume, calculated before the trials, was 15.5 cc per single swallow.

2. After that subjects performed a trial of continuous drinking of 100 cc of tap water.

The testing was repeated twice. A total of six swallows and two drinking periods were obtained per participant. Totally 954 swallows and 318 drinking periods were evaluated during this study. The graphic records were then evaluated. Twenty subjects were re-tested a day later to detect intertrial difference for the duration variable. These subjects were chosen randomly (sealed envelope method) from different group. Interjudge reliability was assessed by comparing scores obtained for each swallowing trial for each of the two tasks. Two judges blinded to group assignment were involved and the test observer agreement was good (Kappa coefficient 0.77).

We examined single swallowing and continuous drinking of 100 cc of tap water from an open cup (duration, mean electric activity of muscles, type of sEMG graphic record, and number of swallows). In the act of continuous drinking, the one gulp water intake can be measured by dividing 100 cc into the number of swallows the individual performed and this can be recorded on an sEMG system.

The data were analyzed off-line by computer. All graphic recordings were initially inspected by eye. The data were statistically evaluated by one-dimensional analysis of variance, SPSS, Standard version 10.0.5 (SPSS, Chicago, IL, 1999), and χ^2 ^criterion using 95% confident interval. The level of significance for all analyses was set at p < 0.05. Normalization procedure was performed for electric amplitude records in order to change computer-calculated mean (raw mean) into real mean (raw mean minus the mean resting potential of an actual muscle group covered by skin). Further on in the results only real mean data is introduced. Obtained P values in multiple comparisons were then corrected using correction factor (Bonferroni method) to adjust the P value and reduce error in interpretation of statistics.

## Results

### Single swallow test

**Figure 2:**
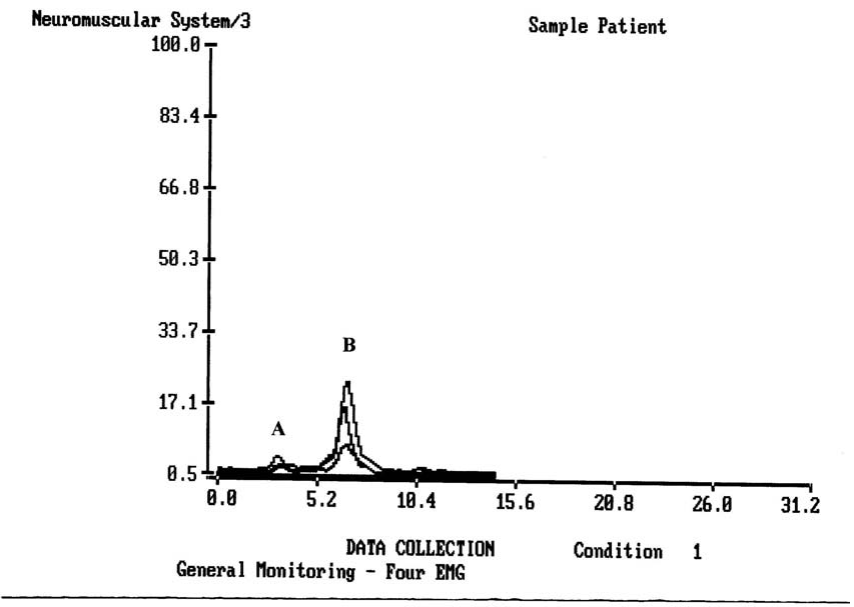
**A typical single water swallow of a healthy person with good coordination of MS, SUB and LSM muscle groups**. Upper peak – SUB location, MS peak slightly precedes SUB peak, and LSM line is the lowest elevation without a clear peak. **A **– lip contact, **B **– a swallow (elevation – pharyngeal stage, drop – beginning of esophageal stage).

**Figure 3:**
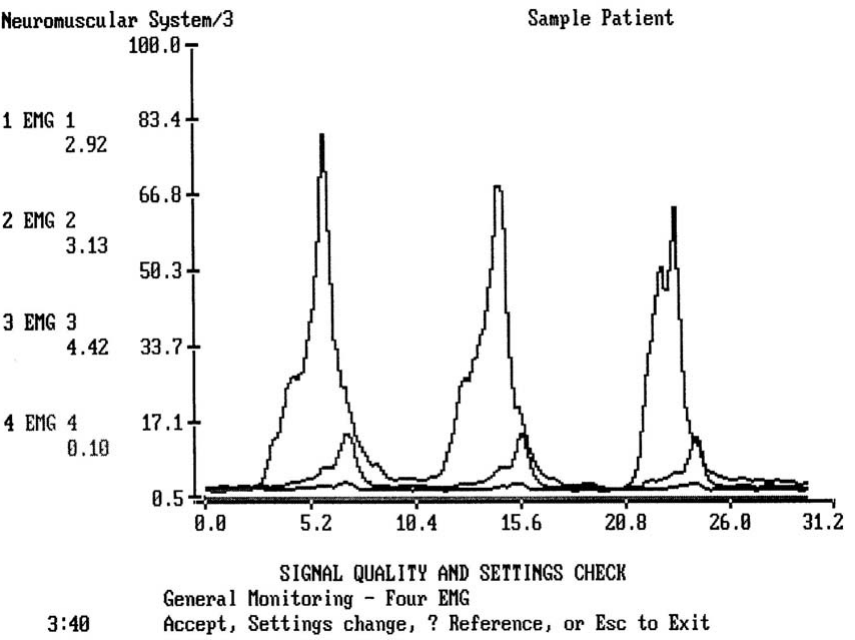
**A typical test of three single normal swallows of a person, 24 hours after dental surgery**. The complete electric activity of muscles during swallow is longer (here 5.21 sec) that normal swallow of a healthy person. The submental and infrahyoid peaks are normal (upper and middle lines), the masseter line (lower line) shows the lack of peaks. The Masseter almost does not work.

Graphically a typical single water swallow of a healthy individual was observed at the rectified and low-passed filtered sEMG as a wave with upward deflections and sharp apex when recorded from both masseter and submental-submandibular locations (Fig. 2). The duration of the reflex phase of muscle activity during single swallows (Table 2) showed prolonged swallow in patients of Groups 1 and 2 (Fig. 3) and normal timing in patients with acute tonsillitis (Group 3, no significant difference with Group 4). This tendency was statistically significant if compared to the healthy volunteers (up to p < 0.01, condition × duration). These times represent the duration of sEMG activity which lasts longer then actual time required to pass a bolus from the oral cavity to the esophagus.

**Table 2:** Single swallow: duration of muscle activity excluding the initial oral stage (mean ± SD)

Dental surgery	Oral infections	Tonsillitis	Control
Group 1	Group 2	Group 3	Group 4

6.41 ± 0.78	4.57 ± 0.61	3.43 ± 0.49	3.35 ± 0.4 sec
p < 0.01	p < 0.05	p > 0.5	

The data for mean and range of EMG activity of masseter, infrahyoid and submental-submandibular muscles during single swallow test are shown in the Table 3. In this test, the mean activity at submental location was 30–50% higher than the activity at masseter location for control group, and 85–95% higher for the group of dental patients, i.e. m. masseter was almost inactive after dental surgery and significantly less active in cases of oral infections. The INF location showed no significant tendency in Groups 1 and 2 but was significantly higher in the acute tonsillitis group (Group 3). In the Group 1, the electric activity of masseter (range, in μV) recorded 72 hours after dental surgery was 35% higher than the activity recorded 24 hours after the surgery. The activity of submental-submandibular and infrahyoid muscles remained the same.

**Table 3:** Electric activity of m. masseter, submental-submandibular and infrahyoid muscles in a single swallow test, in μV (mean* ± standard deviation).

	Dental surgery	Oral infections	Tonsillitis	Control
	Group 1	Group 2	Group 3	Group 4

**Masseter**	9.115 ± 4.2	11.25 ± 5.37	28.33 ± 6.74	17.454 ± 7.55
**Submental**	30.750 ± 9.34	31.63 ± 8.35	34.2 ± 8.54	36.75 ± 10.34
**Infrahyoid**	14.765 ± 3.12	14.422 ± 4.65	36.73 ± 9.62	15.773 ± 6.95

* Real mean is displayed (raw computer-calculated mean minus the mean resting potential of an actual muscle covered by skin (2.808 μV = for the submental location and infrahyoid location, 2.495 μV = for the masseter location), further normalized.

The mean VAS scores during this test were 5.5 for Group 1, 3.6 for Group 2, and 6.2 for Group 3. Correlation between VAS data and sEMG data was insignificant for Groups 1 and 2 (p = 0.24 and p = 0.32 respectively) and significant for Group 3 (p < 0.05).

### Continuous drinking test

The mean volume of liquid per swallow during continuous drinking was 14.7 cc for the control group, and only 9.4 cc for the group of operated patients (Group 1). We monitored a clear tendency for a decrease of the mean volume of liquid per swallow for the operated dental patients (p < 0.01) and patients with oral cavity infections (p < 0.05). Acute tonsillitis (Group 3) did not affect the duration of drinking.

For the Groups 1 and 2, we monitored a tendency for an increase of the duration of muscle activity. This tendency was significant if compared with the control group (p < 0.005 for Group 1, p < 0.05 for Group 2). Specifically, the mean duration of drinking of 100 cc of water was 10.8 sec for the control group, and 18.7 sec for Group 1. The number of swallows for drinking 100 cc of water also increased for the patients in Group 2 (p = 0.01) and Group 1 (p < 0.05) and this was accompanied by a simultaneous decrease of amount of water per swallow (p = 0.01 and p < 0.05 subsequently). Duration of muscle activity during the test of continuous drinking of 100 cc of water is shown in Table 4.

**Table 4:** Continuous drinking of 100 ml water – duration of muscle activity excluding the initial oral stage (mean ± SD)

	Dental surgery	Oral infections	Tonsillitis	Control
	Group 1	Group 2	Group 3	Group 4

Total duration (s)	17.9 ± 5.32	17.3 ± 5.3	10.8 ± 2.1	10.7 ± 2.81
Number of swallows	10.9 ± 3	9.4 ± 2.2	7.1 ± 1.4	6.9 ± 1.82
Duration of one swallow (*s*)	1.64 ± 0.6	1.75 ± 0.7	1.52 ± 0.3	1.51 ± 0.41
ml/swallow	9.17 ± 2.85	10.64 ± 2.4	14.08 ± 3.1	14.5 ± 3.55
ml/swallow in %	69%	73%	97.25%	100%

**Figure 4:**
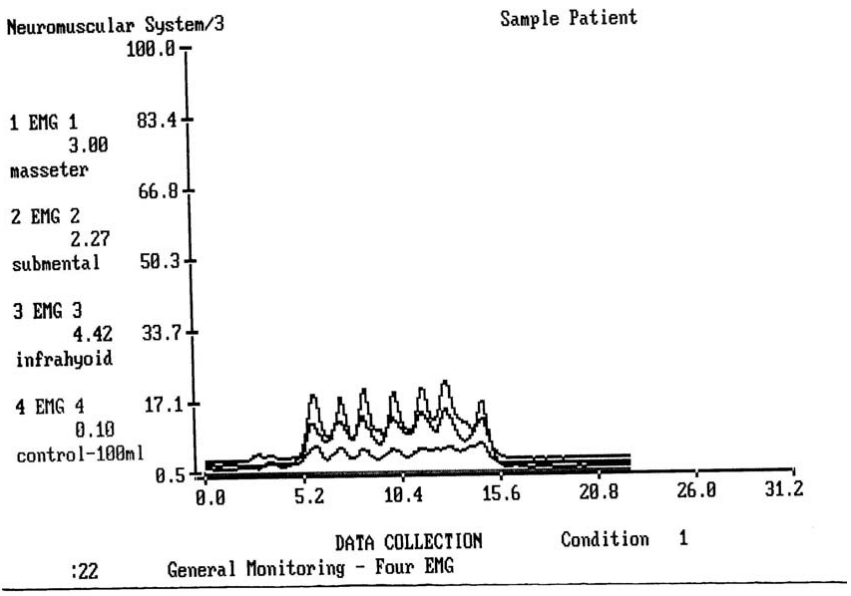
**An example of normal drinking of 100 cc of water**. It took this subject 11.04 sec to drink 100 cc of water in 7 swallows. Upper line = the submental-submandibular electrode location, middle line = the masseter electrode location, lower line = the infrahyoid location. All these muscles are almost completely relaxed before and after drinking.

**Figure 5:**
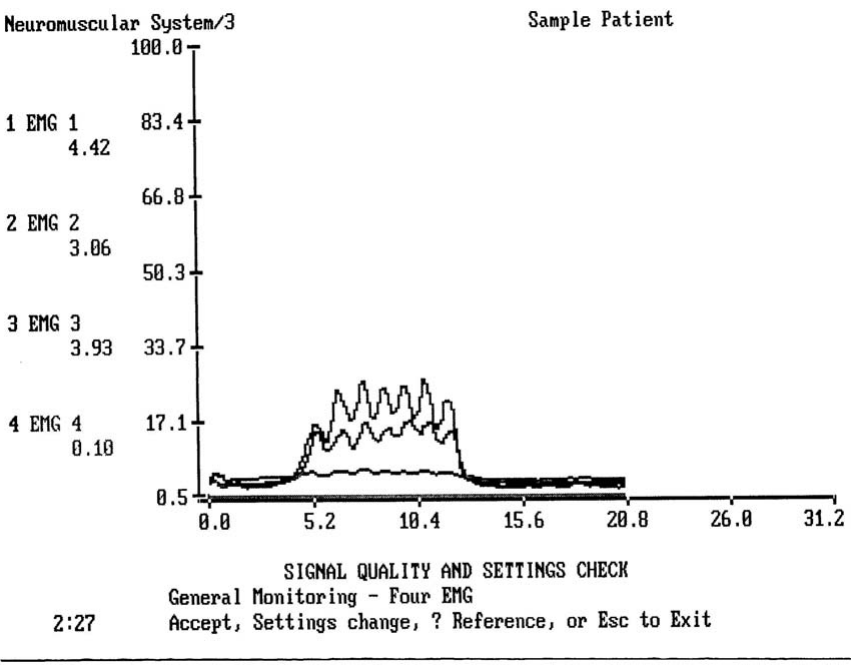
**An example of drinking of 100 cc of water 24 hours after a dental surgery**. Upper line = the submental-submandibular electrode location, middle line = the infrahyoid electrode location, lower line = the masseter location. The masseter line shows no peaks and no elevation during drinking.

In healthy painless drinking, the submental-submandibular location amplitude of electric activity was insignificantly higher than the amplitude of the m. masseter (Fig. 4). In the Group 1 and Group 2 the amplitude of m. masseter was so low, that this difference became significant (p < 0.001) (Fig. 5). The infrahyoid location was less informative for comparison dental patients and controls but became a prominent factor when patients with tonsillitis were compared to the controls. Among the patients in Group 3 the masseter and the infrahyoid electric activities were significantly higher compare to the controls and dental patients (p < 0.05 and p < 0.01 respectively).

The mean VAS scores during this test were 5.1 for Group 1, 3.3 for Group 2, and 5.4 for Group 3. Correlation between VAS data and sEMG data was insignificant for Groups 1 and 2 (p = 0.3 and p = 0.48 respectively) and significant for Group 3 (p < 0.05).

There was no statistically significant gender related difference for the duration and mean muscle activity during both tests for all groups (p = 0.12).

## Discussion

In the current article we want to draw attention on frequently overlooked oral cavity problems as a possible cause for dysphagia. The reason for presenting this study arose from our routine practice in the outpatient clinics of our ENT and Oral and Maxillofacial Surgery departments. We repeatedly consulted patients with dysphagia complaints referred to otolaryngology specialists by general practitioners who appeared to have oral problems. While patients with acute tonsillitis are frequently admitted to outpatient departments, the differential diagnosis between oral and pharyngeal dysphagia should be warranted. The reason for this is that patients seldom present their complaints presicely. A patient after a dental extraction, for example, can say that he/she feels "something uncomfortable" in the throat.

Surface EMG of swallowing is a simple and reliable method for evaluation of single swallowing and continuous drinking with low level of discomfort of the examination [24]. In our departments, the sEMG is used for rapid evaluation of patients' complaints on dysphagia as well as to localize where the pain comes from. For example, in case of dysphagia and/or odynophagia caused by odontogenic infection in the submandibular space, the abnormal records will be observed at SUB location rather than at MS location. The onset of water swallow is usually seen as a mild elevation of the line and represents the final oral stage of a swallow which occurs when the tongue is moved so as to squeeze the liquid volume against the hard palate. Submental muscles and masseter support the tongue-induced pressure. At this stage the automatic reflexive gesture of swallowing is triggered. The same record taken from a patient after dental surgery indicated normal waves at submental-submandibular and infrahyoid locations and much lower wave, or almost no activity at all, at the masseter location.

This phenomenon clearly shows that the dysphagia in dental patients is of oral origin and does not affect pharyngeal stage of a swallow. In fact, this dysphagia is secondary to odynophagia. More precisely, what we might actually had been observing was compensation in patients with painful swallow. A patient tries to spare the operated site, does not clench teeth and, therefore, does not involve masseter in the acts of swallowing and drinking as it was clearly seen at the sEMG records. This condition may be called as "normal swallow modified by pain" (VAS pain scores 6.2–3.6) but terminologically we obliged to label it as dysphagia. Patients with acute tonsillitis might be suspected in having impaired submental muscular activity. In our study, however, the submental activity remained within normal range despite the fact that pharyngeal muscles are the main group of muscles involved in normal deglutition during pharyngeal phase. It seemed that during episodes of tonsillitis abnormally active infrahyoid muscles tried to "assist" the submental muscles in deglutition.

Oral alterations may occur in the occlusion of the teeth following dental surgery, and we may speculate that these might occur either as a direct involvement of the mandibular suspensory apparatus during the surgery, or as a result of compensatory muscle changes. The patients suffering from this condition appear to restore their occlusion to normal, provided that post-surgical recovery is successful. However, there seems to be a sort of break-point at about two weeks post-surgery. If the oral pain persists this long before it subsides, then the masseter seems to stay out of alignment. From the prospective of neuromuscular compensation, if there are functional connections between cervical afferents and V spinal neurons responsible for oral functions, then since most of V spinal neurons appear to be inhibitory to the jaw elevator muscles, one would expect cervical afferent stimulation to have similar effect. In fact, this exactly is seen at the sEMG records. These interactions, however, have yet to be studied.

Surface EMG appeared to be very convenient tool for assessment of patients with both the oral and the pharyngeal dysphagia/odynophagia. Its sensitivity and specificity was proven in several previous works [21-26]. Since the surface EMG monitors the summed activity of large groups of muscle fibers, surface EMG recordings are repeatable. Surface EMG is thus more valid than needle EMG for assessing and quantifying whole muscle function according to statistical criteria [27].

We present a combined sEMG differential diagnosis table that summaries our findings (Table 5).

**Table 5:** Quick reference sEMG differential diagnostic table Oral vs. Pharyngeal Dysphagia.

Condition	Duration	Electric amplitude		
		Masseter	submental	infrahyoid

Dental surgery	prolonged	low	normal	normal
Oral infection	prolonged	low	normal	normal
Acute tonsillitis	normal	high	normal	high

## Conclusion

The duration of the reflex phase of swallows shows significant increase in patients who presented symptoms of dysphagia after lower molar surgery or oral infections. Electric activity of the oral muscles is low in these cases, while activity of submental and infrahyoid muscles remained normal. The timing of events and amplitude data can be used for diagnostic and differential diagnostic purposes, objectivization of complaints, as well as for comparison purposes in pre- and postoperative stages, monitoring of postsurgical healing, tracing postsurgical complications and in EMG monitoring during dental treatment.

## Competing interests

The authors declare that they have no competing interests.

## Authors' contributions

MV designed the concept of the study, contributed in acquisition of data (ENT section), analysis and interpretation of the data, writing. ON contributed in acquisition of data (maxilla-facial surgery section), analysis and interpretation of the data, writing.

## Pre-publication history

The pre-publication history for this paper can be accessed here:


